# Neural Plasticity Induced by Hearing Aid Use

**DOI:** 10.3389/fnagi.2022.884917

**Published:** 2022-05-19

**Authors:** Hanin Karawani, Kimberly Jenkins, Samira Anderson

**Affiliations:** ^1^Department of Communication Sciences and Disorders, Faculty of Social Welfare and Health Sciences, University of Haifa, Haifa, Israel; ^2^Walter Reed National Military Medical Center, Bethesda, MD, United States; ^3^Department of Hearing and Speech Sciences, University of Maryland, College Park, College Park, MD, United States

**Keywords:** age-related hearing loss, auditory processing, amplification, cortical auditory evoked potentials, plasticity, hearing aids, older adults

## Abstract

Age-related hearing loss is one of the most prevalent health conditions in older adults. Although hearing aid technology has advanced dramatically, a large percentage of older adults do not use hearing aids. This untreated hearing loss may accelerate declines in cognitive and neural function and dramatically affect the quality of life. Our previous findings have shown that the use of hearing aids improves cortical and cognitive function and offsets subcortical physiological decline. The current study tested the time course of neural adaptation to hearing aids over the course of 6 months and aimed to determine whether early measures of cortical processing predict the capacity for neural plasticity. Seventeen (9 females) older adults (mean age = 75 years) with age-related hearing loss with no history of hearing aid use were fit with bilateral hearing aids and tested in six testing sessions. Neural changes were observed as early as 2 weeks following the initial fitting of hearing aids. Increases in N1 amplitudes were observed as early as 2 weeks following the hearing aid fitting, whereas changes in P2 amplitudes were not observed until 12 weeks of hearing aid use. The findings suggest that increased audibility through hearing aids may facilitate rapid increases in cortical detection, but a longer time period of exposure to amplified sound may be required to integrate features of the signal and form auditory object representations. The results also showed a relationship between neural responses in earlier sessions and the change predicted after 6 months of the use of hearing aids. This study demonstrates rapid cortical adaptation to increased auditory input. Knowledge of the time course of neural adaptation may aid audiologists in counseling their patients, especially those who are struggling to adjust to amplification. A future comparison of a control group with no use of hearing aids that undergoes the same testing sessions as the study’s group will validate these findings.

## Introduction

Aging can lead to sensory impairments such as age-related hearing loss, which is one of the most common sensory deficits in older adults (Vos et al., 2015; James et al., 2017; Haile et al., 2021). The global burden study (Haile et al., 2021) anticipates that by the year 2050, 698 million people will have moderate-to-profound hearing loss that could benefit from rehabilitation services, and approximately 66% of older adults aged 70 years or older will be reported to have bilateral hearing loss (Collins, 1997; Bowl and Dawson, 2019). This age-related hearing loss may accelerate declines in cognitive and neural function and dramatically affect the quality of life (Heine and Browning, 2002; Lin et al., 2013; Loughrey et al., 2018; Rutherford et al., 2018); indicating a strong need for effective intervention.

Hearing aids are the most common treatment for mild-to-moderate age-related hearing loss. Although hearing aid technology has advanced dramatically over the last decade, the Global Burden of Disease estimates suggest that there is an 83% unmet need for hearing aids globally, calculated as the proportion of individuals with moderate-to-severe hearing loss who do not use a hearing aid (Orji et al., 2020). Our previous findings have shown that the use of hearing aids improves cortical and cognitive function (Karawani et al., 2018a) and offsets subcortical physiological decline (Karawani et al., 2018b). However, many people do not use their hearing aids (e.g., Bisgaard and Ruf, 2017; Hoppe and Hesse, 2017; Johnson et al., 2018), and one of the reasons for their lack of use is that they have difficulty adjusting to amplified sounds (Johnson et al., 2018). A better understanding of the hearing aid adjustment process and the time course of adaptation may lead to more effective management of hearing loss.

New hearing aid users require time to become accustomed to their hearing aids (e.g., Cox and Alexander, 1992; Gatehouse, 1992; Horwitz and Turner, 1997; Kuk et al., 2003; Munro and Lutman, 2003; Yund and Woods, 2010; Giroud et al., 2017; Karah and Karawani, 2022). Evidence supporting auditory adaptation with hearing aids is mixed, and the extent to which the auditory system adapts to new input remains unknown. The current study aims to test the time course of this adjustment period and to determine how objective measures can be used to provide information regarding potential hearing aid outcomes that can be used in the adaptation period. These aims will be accomplished by examining neuroplastic changes over the course of 6 months in newly fit hearing aid users.

Several studies have examined perceptual adaptation in older adults who were first-time hearing aids users, but they have had differing conclusions. Some studies show significant improvement in perceptual measures over time (Gatehouse, 1992; Horwitz and Turner, 1997; Munro and Lutman, 2003; Reber and Kompis, 2005; Munro, 2008; Olson et al., 2013; Lavie et al., 2015; Dawes and Munro, 2017; Karawani et al., 2018b; Karah and Karawani, 2022). For example, Gatehouse (1992) and Munro and Lutman (2003) tested older adult participants with sensorineural hearing loss who were fit with hearing aids monaurally. Following a period of 12 weeks of monaural hearing aid use in four participants, Gatehouse reported that aided speech recognition improved in the fitted ear but not in the unfitted ear. Munro and Lutman (2003) also observed significant improvements in speech recognition for the fitted vs. unfitted ears in sixteen participants. Reber and Kompis (2005) tested older adults who were fit with bilateral hearing aids at 2 weeks and 6 months after hearing aid use and found improvement in speech-in-noise recognition over time. Lavie et al. (2015) tested older adults after 4, 8, and 14 weeks of hearing aid use and found that unaided dichotic listening scores and unaided speech identification in noise improved significantly after 8 weeks of hearing aid use. Recently, Wright and Gagné (2020) showed an increase in speech in noise performance following 4 weeks of hearing aid use, suggesting an adaptation effect.

While these studies have suggested that adaptation may be observed post-fitting from four to eighteen weeks up to 6 months (e.g., Cox and Alexander, 1992; Gatehouse, 1993; Reber and Kompis, 2005; Giroud et al., 2017; Wright and Gagné, 2020), other studies reported that the effects were minimal (Bender et al., 1993), or not evident at all (Humes and Wilson, 2003; Dawes et al., 2014a). This inconsistency between studies may have been due to design and methodology factors, such as unilateral vs. bilateral fitting, the amount of auditory input and the period of the hearing aid use, and other hearing loss severity and cognitive factors (Palmer et al., 1998) or the timing of the baseline test. For example, Humes and Wilson (2003) tracked speech recognition changes over a 3-year period of bilateral hearing aid use in nine older adults at intervals of 1, 6, 12, 24, and 36 months after hearing aid fitting, and little evidence of speech recognition improvement in aided performance was noted. The initial testing of aided performance was conducted after 1 month of hearing aid use, and any possible gains in performance during the first month (e.g., Dawes and Munro, 2017; Wright and Gagné, 2020) may have limited the potential for further gains. Therefore, the current study aimed to examine effects of hearing aid use by controlling baseline measures on the day of fitting.

In addition to perceptual and behavioral measures, research has been conducted to study neural changes induced by newly fit hearing aids in older adults using subcortical (Philibert et al., 2005; Karawani et al., 2018b) and cortical (McCullagh, 2009; Dawes et al., 2014b; Giroud et al., 2017; Rao et al., 2017; Habicht et al., 2018; Karawani et al., 2018a; Maruthy, 2019; Glick and Sharma, 2020) electrophysiological measures. Other research has been conducted in experienced users (e.g., Gatehouse, 1995; Munro et al., 2007; Bertoli et al., 2011; McClannahan et al., 2019). In the following paragraphs, we focus on previous studies that have evaluated changes in cortical auditory evoked potentials (CAEPs) in new hearing aid users.

The CAEP has been used to examine the effects of auditory stimulation and amplification while wearing hearing aids in normal-hearing and hearing-impaired participants (e.g., Korczak et al., 2005; Billings et al., 2007, 2011; Van Dun et al., 2016; Jenkins et al., 2018). More specific to the current study, CAEPs have been used to examine neural changes in hearing ability following a period of hearing aid use (McCullagh, 2009; Dawes et al., 2014b; Giroud et al., 2017; Rao et al., 2017; Karawani et al., 2018a; Habicht et al., 2018; Maruthy, 2019). CAEPs have relatively high temporal resolution and can provide detailed insights into the neural processing of auditory signals and integrative processing in the auditory cortices (for a review, see Eggermont, 2007).

As mentioned earlier, a number of studies have tracked the results of using bilateral newly fit hearing aids using CAEPs for a period of 4 weeks to 6 months in older adults. Specifically, Rao et al. (2017) studied P3 peak changes using an oddball paradigm to assess neural changes after 4 weeks of hearing aid use and found a significant reduction in P3a amplitude. Giroud et al. (2017) also used an active oddball paradigm, and reported significant reductions in the global field power in the P3b after 3 months of intensive hearing aid use. Habicht et al. (2018) combined electrophysiology (N2 and P3 responses) and eye tracking to compare newly fit hearing aid users with experienced users. The first-time hearing aid users group showed smaller N2 amplitudes than the experienced users at baseline; however, no changes in N2 amplitudes were observed over time (after 24 weeks in the first-time hearing aid group). McCullagh (2009) observed earlier N1 latencies after 6–8 weeks of hearing aid use but did not observe changes in N1 and P2 amplitudes or P2 latency. They suggested that the change observed in N1 latency reflects a physiological adaptation effect.

Karawani et al. (2018a) compared a group of first-time hearing aid users with a hearing-matched control group with no use of hearing aids after a period of 6 months. The use of hearing aids was associated with improvement in working memory performance and increased cortical response amplitudes for the N1 and P2 peaks. The N1 component is believed to reflect early triggering of attention to auditory signals (Näätänen, 1990; Čeponien et al., 2002). Therefore, this finding suggests that increased auditory experience gained through hearing aid use for 24 weeks resulted in greater allocation of attentional resources to the signal. The P2 peak component is believed to reflect auditory object identification (Ross et al., 2013), and changes in P2 amplitudes were positively related to working memory improvement. These amplitude enhancements suggest that hearing aid use may alter cortical processing and reflect a physiological adaptation effect. These results contrast with those of Dawes et al. (2014b) who did not report changes in cortical amplitudes/latencies after hearing aid use, possibly due to differences in stimuli. Dawes et al. (2014b) presented pure-tone stimuli through insert earphones while Karawani et al. (2018a) presented speech stimuli through free-field speakers. Stimulus type might affect the neural encoding in the central auditory system (Tremblay et al., 2004; Billings et al., 2011; Xie et al., 2021), and the P1-N1-P2 complex is sensitive to stimulus characteristics (e.g., Papanicolaou et al., 1984; Ostroff et al., 2003; Michalewski et al., 2005).

Our previous study (Karawani et al., 2018a) showed changes in neural processing after 6 months of hearing aid use. In the current study, we aimed to determine the time course of the changes in CAEP amplitudes noted in that previous study (N1 in quiet, P2 in quiet, and P2 in noise) at six time points: 0, 2, 6, 12, 18, and 24 weeks of hearing aid use. We also aimed to determine if these measures can be used by the clinician to provide information regarding potential adaptation to newly fit hearing aid individuals. We should note that that a control group was tested during the first and last sessions, but the study lacked a control group that underwent testing during the other four sessions.

## Materials and Methods

### Participants

Data from thirty-one older adults (18 females) between the ages of 60–84 years were included in this study. These data were taken from a larger research study (previously published in Karawani et al., 2018a,b) from the Washington D.C. metro area. All participants were native English speakers recruited through printed advertisements in local senior living communities and Craigslist advertisements. The Institutional Review Board of the University of Maryland, College Park approved all procedures. All participants provided written, informed consent prior to participation and received compensation for their time. Participants underwent bilateral audiometric threshold assessment of pure-tone air-conduction (from 250 to 8,000 Hz) and bone-conduction (from 250 to 4,000 Hz) thresholds. They had sensorineural symmetrical hearing loss with no air-bone gaps or asymmetries between ears exceeding 15 dB HL. All participants underwent cognitive evaluation using the Wechsler Abbreviated Scale of Intelligence (Zhu and Garcia, 1999) and had normal IQs (≥85). The Montreal Cognitive Assessment (MoCA) was used to screen for mild cognitive impairment (Nasreddine et al., 2005), a cutoff of 22/26 was used as suggested by Dupuis et al. (2015) for individuals with hearing loss. Participants had no history of neurological or psychiatric diseases, were native English speakers (with no report of bilingualism), and had no significant history of musical training.

**FIGURE 1:**
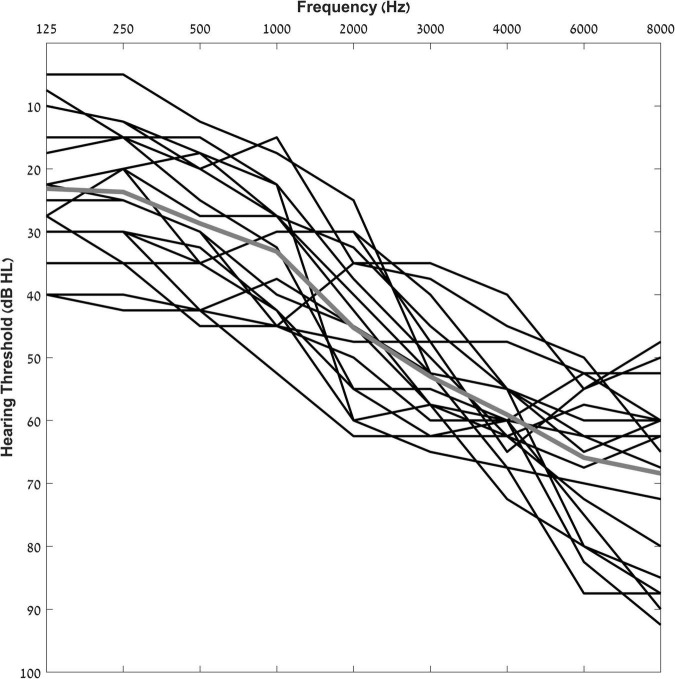
Individual pure-tone air-conduction thresholds for participants from 125 to 8,000 Hz. The solid gray line indicates group average pure tone thresholds.

Participants that met the inclusion criteria listed above were fit with bilateral hearing aids and were seen in several testing sessions during a period of 6 months. The final number of participants that completed all six sessions and were included in the experimental group of the current study was 17 (9 females, mean age = 75 years ± 6); their audiograms are shown in Figure 1.

### Study Design

**FIGURE 2:**
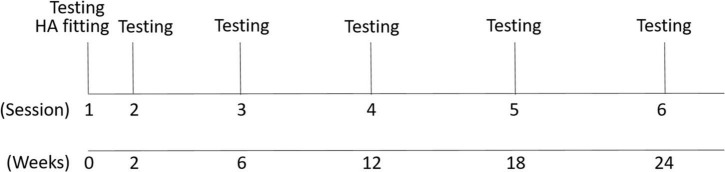
Study design. Six testing sessions were conducted. The first session included the hearing aid fitting and EEG recording and the second to sixth sessions (EEG recording and hearing aid checks of data logging) were conducted after the hearing aid fitting in time intervals shown in weeks.

All participants in the experimental group were fit with bilateral hearing aids and tested in six testing sessions as shown in Figure 2. Hearing aids were fit at the first session, and the participants returned for follow-up testing at 2, 6, 12, 18, and 24-week intervals. Electrophysiological testing was conducted at each session.

**FIGURE 3:**
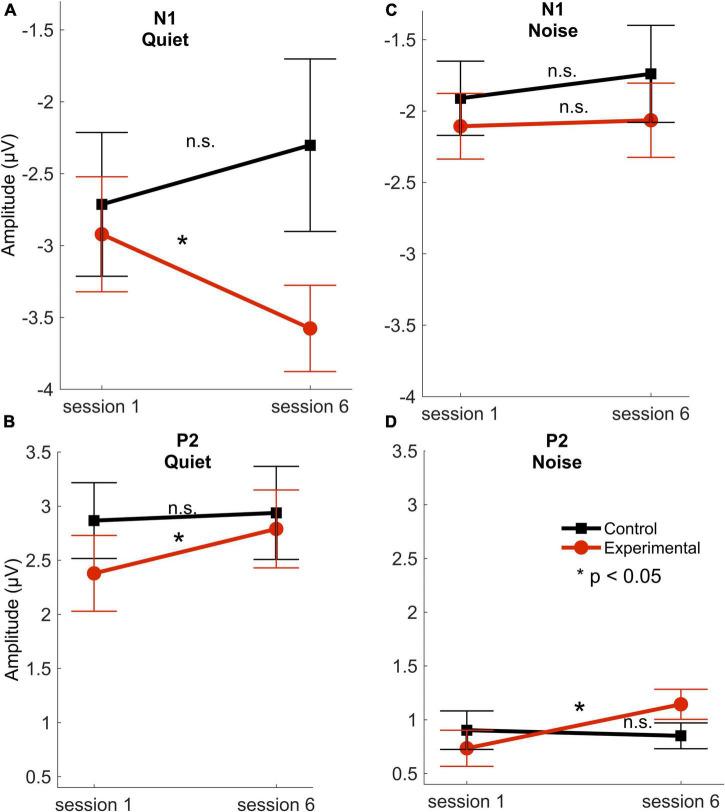
Response amplitudes. N1 and P2 mean amplitudes for the experimental (red circles) and control (black squares) groups across sessions 1 (first) and 6 (last) in quiet **(A,B)** and noise **(C,D)** conditions. Error bars represent standard error of the mean. *P < 0.05. n.s, not significant. There were no significant differences between the control and experimental groups in session 1 in any of the cortical amplitudes in quiet [N1: *t*(29) = 0.665, *p* = 0.511; P2: *t*(29) = 1.331, *p* = 0.194] or in noise [N1: *t*(29) = 0.597, *p* = 0.555; P2: *t*(29) = 0.505, *p* = 0.617] (modified with permission from Karawani et al., 2018a).

Data from a control group who underwent identical testing sessions in sessions one and six were used in the analysis to ensure that there were no cortical changes between these sessions. These control data from the first and sixth sessions were published in Karawani et al. (2018a). The control group consisted of 14 participants (9 females; mean age 74 ± 6) and were fit with bilateral hearing aids during the two testing sessions, but they did not use any hearing aids through the period between sessions one and six. The control group serves as a comparable group to the experimental group in these demographic factors (presented in Table 1): age, gender, pure-tone average hearing and high-frequency hearing, IQ, and MoCA scores; *p* > 0.08. The control group’s data analysis showed that there was no cortical change in amplitudes of the peaks P1, N1, and P2 between sessions 1 and 6 in quiet [P1: *t*(13) = 0.236, *p* = 0.816; N1: *t*(13) = 1.102, *p* = 0.290; P2: *t*(13) = 0.238, *p* = 0.816] or in noise [P1: *t*(13) = 0.769, *p* = 0.455; N1: *t*(13) = 0.527, *p* = 0.607; P2: *t*(13) = 0.425, *p* = 0.678]. In addition, there was no significant change in latencies of the peaks P1, N1, and P2 between sessions 1 and 6 in quiet [P1: *t*(13) = 0.265, *p* = 0.795; N1: *t*(13) = 0.676, *p* = 0.511; P2: *t*(13) = 1.940, *p* = 0.075] or in noise [P1: *t*(13) = 1.946, *p* = 0.074; N1: *t*(13) = 0.689, *p* = 0.503; P2: *t*(13) = 0.123, *p* = 0.240]. There were no significant differences between the control and the experimental group in session 1 in any of the cortical components across conditions [*t*(29) < 1.087, *p* > 0.285] (Figure 3). The sections below refer to the analysis conducted for the seventeen participants in the experimental group.

**TABLE 1:** Demographics.

	Experimental	Control	*t*(29)	*P*-value
*N*	17	14		
Age range (years)	60–84	62–84		
Age (years)	75.41 (6.71)	73.71 (5.79)	0.739	0.466
Male/female	8/9	5/9	1.036	0.309
Pure-tone average hearing (0.5–4 kHz; dB HL)	42.20 (7.18)	40.21 (8.37)	0.713	0.482
High-frequency hearing (6–8 kHz; dB HL)	65.58 (11.54)	60.98 (13.47)	1.026	0.314
IQ	114.05 (9.32)	112.72 (6.94)	1.673	0.120
MOCA	26.88 (1.69)	25.24 (2.45)	1.811	0.088

*Groups were matched on all demographic factors. Means (SDs) are displayed for age, sex distribution, hearing, IQ, and Montreal Cognitive Assessment (MoCA) scores. Number of participants in each group (N), t-values with degrees of freedom and P-values of the group comparison are also shown.*

### Hearing Aid Fitting

The hearing aid fitting procedure was previously described in Karawani et al. (2018a). Receiver-in-the-canal Widex Dream 440 hearing aids were used for bilateral fitting. The hearing aids had size M receivers (to ensure accommodation to hearing losses up to 85 dB HL from 125 to 8,000 Hz) and domes most appropriate for their hearing loss, (open: thresholds for 250–500 Hz < 30 dB HL; tulip: individual thresholds for 250–500 Hz ≥ 30 dB HL). The hearing aid fitting was performed immediately following the audiologic examination on the first day of testing. For the purpose of this study, a single automatic program was used. In addition, the participants did not have the opportunity to alter the hearing aid gain. Real-ear measurements were performed to verify the fitting (for more details please refer to Karawani et al., 2018b). Most of the output values met the Goodness of Fit test (*F* > 5.315, *p* < 0.030, *R*-squared > 0.181). Maximum power output measurements were conducted to ensure that the hearing aids did not exceed maximum tolerance limits. On the first day, the participants received an in-service on hearing aid use and were instructed to begin wearing their hearing aids at least 8 h per day. Participants were advised that the hearing aids were set according to their audiometric thresholds, and that for the purposes of the study aims, no changes could be made to the settings. They were also told that they would adjust to the prescribed amplification if they wore their hearing aids on a daily basis. Upon request, at the end of 6 months, changes were made to features such as gain, amplification, directionality, etc. To ensure compliance, monitoring of hearing aid use (average hours/day) was done at each follow-up session through the hearing aid data logging function available through the Widex software platform (group average = 9.31 h/day ± 2 h).

### Cortical Auditory Evoked Potentials

All tests were conducted in an electrically-shielded sound-attenuated booth. Participants wore their hearing aids during the recording session and were seated in an upright position at a distance of two meters from an Interacoustics SP90 speaker at 0° azimuth (as described in Karawani et al., 2018a,b). This seating position was identical in all testing sessions. During the recordings, participants watched a silent, closed-captioned movie of their choice to facilitate a relaxed but wakeful state. Cortical auditory-evoked potentials (CAEPs) were recorded to a 170-ms speech syllable/ga/presented through the Interacoustics speaker via Presentation software (Neurobehavioral Systems, Inc.) in two listening conditions: (1) 80 dB SPL in quiet (referred to as quiet condition) and (2) 80 dB SPL in the presence of 70 dB SPL 6-talker babble [+ 10 dB Signal-to-noise ratio (SNR), noise condition]. The 6-talker babble was taken from the Words-in-Noise sentence lists (Wilson et al., 2003) and was continually looped every 4.6 s. For more specific details of stimulus features, please refer to Karawani et al. (2018a). A Larson Davis System 824 sound level meter was used to perform calibration prior to each session to ensure that the /ga/and noise stimuli were within ± 1 dB of the stimulus level at ear level.

#### Recording

The Biosemi Active-Two acquisition system (BioSemi B.V., Amsterdam, Netherlands) was used to record responses at a sampling frequency of 2,048 Hz via a 32-channel electrode cap. The offsets for all channels were below 50 μV, and earlobes served as references. Six hundred artifact-free sweeps were collected for each condition.

#### Data Processing and Analyses

MATLAB (MathWorks, version R2011b) was used for offline processing. Zero-phase offline bandpass filtering was performed from 1 to 30 Hz, using a 4th-order Butterworth filter. An electro-oculography reduction method (Romero et al., 2006; Schlögl et al., 2007) was used to remove eye movements. Each sweep consisted of a time window of −100 to 400 ms with respect to the stimulus onset. The offline artifact-reject criterion was set at ± 100 μV. The final average response was composed of the first 500 artifact-free sweeps.

#### Data Analysis

Karawani et al. (2018a) found significant amplitude increases for N1 in quiet and for P2 in quiet and in noise; therefore, in the current manuscript, we limited our analyses to these components. An automated peak-peaking algorithm in MATLAB was used to calculate mean response amplitudes from the Cz electrode for the expected time regions of each of the dominant cortical peaks: N1 (80–150 ms) and P2 (160–250 ms) in the quiet condition, and N1 (150–200 ms) and P2 (225–275 ms) in the noise condition. The test-retest reliability of the CAEP amplitudes is moderate relative to brainstem amplitudes (Bidelman et al., 2018). Because we wanted to maximize test-retest reliability, we chose to measure changes in the Cz amplitude, which is more robust in quiet and noise (Papesh et al., 2015).

### Statistical Analysis

*Changes across six sessions*: Repeated measures analyses of variance (RMANOVA) were performed using time (6 sessions) as a within-subject factor for amplitude and latency of peaks N1 (in quiet and in noise) and P2 (in quiet and in noise), followed by planned paired *t*-tests. *Predictive measures*: As stated in the introduction, we also aimed to determine if these amplitude measures can be used by the clinician to provide information regarding potential adaptation to newly fit hearing aid individuals. Therefore, regression model analysis was conducted to determine whether earlier sessions predicted improvement in the final session. *Confidence intervals* (Lambert et al., 1991): We calculated the confidence intervals for amplitudes across participants at session 1, and then used this measure as a criterion to determine the presence of singificant amplitude changes in individual participants at each follow-up session.

## Results

### Changes Across Six Sessions

**FIGURE 4:**
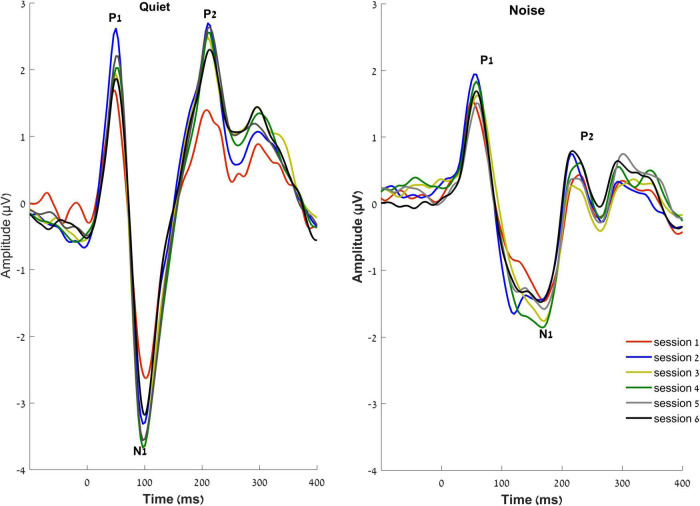
Grand averaged waveforms of the experimental group at the vertex electrode Cz, in quiet **(right)** and noise **(left)** conditions over the six sessions.

**FIGURE 5:**
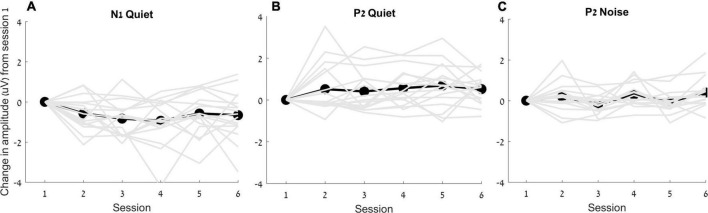
Change in amplitudes (in μV) from the first session for N1 in quiet **(A)**, P2 in quiet **(B)** and P2 in noise **(C)** are plotted for each individual (gray lines) as well as for the average (black lines).

The RMANOVA showed a main effect of time [*F*_(5, 16)_ = 2.055, *p* = 0.006, η^2^*_*p*_* = 0.128]. *Post hoc* pairwise comparisons between sessions after adjusting for multiple comparisons (Benjamini and Hochberg, 1995) showed that changes from session 1 were observed earlier for N1 amplitude than those for P2 amplitude, such that N1 amplitude in quiet increased as early as 2 weeks after the hearing aid fitting (*p* = 0.031), but P2 amplitude in quiet did not increase until 6 weeks after hearing aid fitting (*p* = 0.033). Furthermore, a significant increase in P2 amplitudes in noise was observed in the final session—24 weeks following the initial hearing aid visit (*p* = 0.012) (Figures 4, 5), but N1 in noise amplitudes did not show any significant changes (*p* > 0.3). Taken together, these results demonstrate evidence of neuroplasticity in N1 amplitudes earlier (2 weeks following hearing aid fitting) than P2 amplitude changes (6 weeks after hearing aid fitting). The time course of neuroplasticity during the period of hearing aid use is reflected in Figure 5. For each participant, amplitude values were adjusted such that session 1 (day 1) values were fixed to 0, then, for each subsequent session, amplitude values were presented as the difference (in μV) from session 1. The RMANOVA was also conducted on the peak latency values and no main effect of time was observed [*F*_(5, 16)_ = 1.156, *p* = 0.334], consistent with our previous finding of no change in latency between sessions 1 and 6.

### Predictive Measures

We tested whether changes in N1 amplitude that were observed in the earlier sessions predicted the increase in amplitude in the final session (week 24), thus providing a prognosis for eventual hearing aid improvement. Therefore, we examined the amplitude of N1 in quiet at the first session along with the change in amplitude in session 2 and session 3; i.e., 2, and 4 weeks after fitting, to determine whether clinicians can use these measures in these early sessions to provide the patient with expectations regarding the adaptation process. The “Enter” method of linear regression was used since it specifies the order of variables in the model, and the independent variables were entered in the following order: amplitudes at session 1, change in amplitude at session 2 and change in amplitude at session 3, with change in amplitude at session 6 as the dependent variable. To rule out strong correlations between predictor variables, collinearity diagnostics were performed showing satisfactory variance inflation factor (highest = 1.30) and tolerance (lowest = 0.76) scores. The analysis showed that the earlier sessions highly predict variance in the sixth session (*R*^2^ = 0.655, *p* = 0.004) (Table 2). Of the three measures, the amplitude change at session 3 was the only variable that significantly predicted change at session 6 (*p* = 0.01).

**TABLE 2:** Regression model: Early sessions predicted neuroplastic changes after 6 months of hearing aid use.

	*R*	*R*_change_^2^	F_change_	df1,df2	*p*
**Model**	0.809	0.655	7.589	3,13	0.004

**Variables**				**β**	

Amplitude (session 1)				0.192	0.34
Change in amplitude (session 2–session 1)				0.362	0.05
Change in amplitude (session 3–session 1)				0.576	0.01

*R for full model and the change in R-squared and statistics (following the addition of the change in the later sessions), F-values with degrees of freedom and p-values are presented. Standardized (β) coefficients and significance (p-values) in each variable’s contribution to the model is also presented for the N1 amplitudes collected in sessions 1, the change in N1 amplitude observed in sessions 2 and session 3.*

### Confidence Intervals

The 85% confidence intervals were calculated for session 1 across measures to determine the criterion for significant amplitude change in an individual (see Table 3). This percentage was chosen rather than the conventional 95% confidence interval due to our relatively small sample size and preliminary nature of the study (Lee et al., 2014). For N1 amplitudes in quiet the criterion for significant change was 0.61 μV, for P2 amplitude in quiet the criterion was 0.50 μV, and for P2 amplitude in noise the criterion was 0.25 μV. The results show that for N1 amplitude in quiet, 47% of the participants demonstrated a significant increase after 2 weeks (i.e., 47% had a change larger than 0.61 μV) and 64% demonstrated a significant increase after 6 weeks (Table 4). For P2 amplitude in quiet, 50% of the participants demonstrated a significant increase after 12 weeks, and for P2 amplitude in noise, improvement in 50% of more of the participants was not observed until 24 weeks of the use of hearing aids.

**TABLE 3:** Confidence interval of the difference.

	85% Confidence interval		
Session 1	of the difference		
	Lower	Upper	Difference from the mean (μV)
N1 in quiet	−3.77	−2.53	0.61
P1 in quiet	1.71	2.72	0.50
P2 in noise	0.489	0.99	0.25

*Values presented for session 1 across the three components: N1 in quiet, P2 in quiet and in noise.*

**TABLE 4:** Percentage of the individuals that met the clinical criteria of improvement.

	Session 2	Session 3	Session 4	Session 5	Session 6
N1 in quiet	47%	65%	59%	53%	48%
P1 in quiet	35%	41%	41%	62%	56%
P2 in noise	41%	24%	47%	18%	53%

## Discussion

The main goal of the present study was to determine the time course of neural adaptation to hearing aids and to determine whether early measures of cortical processing predict the capacity for neural change. The current study tested neuroplastic changes induced by hearing aid use over the course of 6 months using CAEPs. Neural changes were observed as early as 2 weeks following the initial fitting of hearing aids. The results showed a neural relationship between responses in earlier sessions and the change predicted after 6 months of the use of hearing aids. These results are significant, because a period of 4 weeks is usually provided for adjustment to the hearing aids, during which the patient can decide whether or not to keep the hearing aids, and knowledge of the potential for neural adaptation may be useful in the decision making process.

A previous study by the authors (Karawani et al., 2018a) reported significant improvements in CAEPs following 24 weeks of hearing aid use. The current study suggests that increases in N1 amplitude can be observed as early as 2 weeks following hearing aid fitting, whereas P2 amplitudes appear to require a longer time course of 6 weeks to observe similar amplitude increases. In the following paragraphs, we discuss a possible interpretation of this finding by considering the generators and mechanisms of both components.

P2 appears to reflect stimulus detection and identification, based on the spectral information provided by temporal-lobe generators, specifically located in auditory cortices of Heschl’s gyrus (e.g., Lütkenhöner and Steinsträter, 1998). N1 generators were shown to provide sound feature specifics and serve the pre-attentive detection of auditory events (Näätänen, 1990). Therefore, N1 might contribute to the processing of word onsets (e.g., Eggermont and Ponton, 2002) and phonetic structure (e.g., Sanders and Neville, 2003) during the perception of continuous speech, and reflects sensitivity to sound audibility (Martin et al., 1997) and early triggering of focused attention to the incoming auditory stimuli (Čeponienë et al., 2008). P2 generators might have access to more fine-grained spectral stimulus information than the N1 generators (Čeponienë et al., 2008), and therefore P2 peak has been shown to reflect integration of stimulus features to facilitate auditory object representation and stimulus identification (Näätänen and Winkler, 1999; Čeponienë et al., 2008; Ross et al., 2013). This level of resolution was reflected in the findings of the current study. It appears that stimulus detection improves rapidly (N1 amplitude changes) after increased audibility through hearing aid use, but a longer period of adaptation to hearing aids is required for identification and assignment of relevance of the stimulus (reflected by P2). Therefore, increased audibility through hearing aids may facilitate rapid increases in cortical detection, but a longer time period of exposure to amplified sound may be required to integrate features of the signal and form auditory object representations.

We note that significant increases in P2 amplitudes were observed only after session 4. Therefore, it is less likely that P2 amplitudes were affected by increased stimulus exposure or repeat testing effects, at least in the early weeks of the study, as has been reported in previous auditory training studies in normal-hearing young adults (Tremblay et al., 2010, 2014). Based on these studies, we might have expected to find P2 but not N1 changes at the second and third follow-up visits. We believe that the differences in findings may arise from differences in hearing ability between groups. Perhaps older adults with hearing loss require more experience with audible sound to show changes in cortical processing. Therefore, the changes we found for the P2 component in older adults with hearing loss may be a potential indicator of later adaptation to hearing aids, as discussed above.

As mentioned in the introduction, studies have shown mixed results concerning neural adaptation at subcortical (e.g., Philibert et al., 2005; Habicht et al., 2018) and cortical levels (e.g., Billings et al., 2007; Bertoli et al., 2011; Dawes et al., 2014b; Dawes and Munro, 2017; Giroud et al., 2017; Karawani et al., 2018a). The current study demonstrates rapid neural adaptation to hearing aids using CAEPs in older adults. Giroud et al. (2017) also reported changes in brain activity after the use of hearing aids for 12 weeks. Maruthy (2019) also showed evidence for neural plasticity and hearing aid benefits after 1 month of hearing aid use, using methods similar to those in the present study (CAEPs) but in younger to middle-aged adults (ages 23–60 year). They reported earlier P1 and N1 latencies after 4 and 8 weeks following the initial fitting of hearing aids. Other research has also documented neural adaptation using other electrophysiological measures. A recent study using cortical visual evoked potentials reported that after hearing aid use for a period of 6 months, reduced cortical activation in temporal and frontal regions with increased activation in visual regions were observed for visual stimuli processing (compared to baseline non-hearing aid use), suggesting neural plastic changes in the cortex after the use of hearing aids for 6 months (Glick and Sharma, 2020). Using Functional magnetic resonance imaging (fMRI), Yu et al. (2017) reported neural changes assessed by fMRI in a clinical case study. An older adult with bilateral sensorineural hearing loss was fit for the first time with hearing aids and was tested at baseline and after 8 weeks. After 8 weeks of hearing aid use, increased responses to audio-visual stimulation was observed, specifically in the superior temporal sulcus (STS). They suggest that this increased activation seen in the STS following the use of hearing aids reflects increased phonological representation of speech sounds, and more efficient use of auditory cues due to adaptation to acoustic amplification through hearing aids. Taken together, rapid neural adaptation can be assessed as soon as 2–4 weeks following hearing aid fitting. The combination of electrophysiological and imaging paradigms would be important for further investigation of neuroplastic changes induced by hearing aids.

## Conclusion

Age-related hearing loss is considered one of the most prevalent health conditions in older adults (Yueh et al., 2003). Although hearing aid technology has advanced dramatically over the last decade, less than one quarter of the population of older adults with hearing loss use hearing aids (Popelka et al., 1998; Lin, 2011). This untreated hearing loss may accelerate declines in cognitive (Lin, 2011; Lin et al., 2013; Peelle and Wingfield, 2016) and neural function (Karawani et al., 2018a). The finding of the current study that cortical changes may occur in as little as 2 weeks may provide encouragement regarding the potential for neuroplasticity, and perhaps eventual improvements in perception. Perhaps this knowledge may increase the patient’s willingness to persist with the process of adaptation.

## Limitations

This study was a relatively small study with the aim of providing pilot data for a larger study that includes more participants and a longer adaptation period. Therefore, a larger cohort would be needed to overcome inter individual variability. Due to the limited nature of the study, it was only possible to test the control group at sessions 1 and 6. Therefore, the observed changes in the hearing aid use group may be due to repeated testing effects. Future studies should include a control group that is tested for the same number of sessions, or perhaps a delayed treatment group that includes multiple baselines. Another suggestion for future research is to conduct a similar study design with first time hearing aid users but with a parallel group of experienced users, and for a longer period of auditory rehabilitation. In addition, associating the neural findings with behavioral changes in speech perception is important, and a future study would benefit from the inclusion of perceptual/self-assessment measures at all-time points. Finally, a future study should consider employing an active listening protocol to eliminate possible confounds of watching a subtitled movie on attention-related cortical components.

## Data Availability Statement

The original contributions presented in the study are included in the article/supplementary material, further inquiries can be directed to the corresponding author/s.

## Ethics Statement

The studies involving human participants were reviewed and approved by the Institutional Review Board of the University of Maryland, College Park. The patients/participants provided their written informed consent to participate in this study.

## Author Contributions

HK designed the study, analyzed the data, interpreted the results, and wrote the manuscript. KJ collected the data. SA designed the study, collected the data, interpreted the results, and wrote the manuscript. All authors approved the final version of the manuscript.

## Conflict of Interest

The authors declare that the research was conducted in the absence of any commercial or financial relationships that could be construed as a potential conflict of interest.

## Publisher’s Note

All claims expressed in this article are solely those of the authors and do not necessarily represent those of their affiliated organizations, or those of the publisher, the editors and the reviewers. Any product that may be evaluated in this article, or claim that may be made by its manufacturer, is not guaranteed or endorsed by the publisher.
